# What biomarkers (if any) for precise medicine?

**DOI:** 10.18632/aging.100798

**Published:** 2015-08-29

**Authors:** Sabrina Strano, Paola Muti, Giovanni Blandino

**Affiliations:** Translational Oncogenomic Unit, Regina Elena National Cancer Institute, Rome, Italy

The advent of the *OMIC* technologies has strongly evolved the knowledge about the origin, the type and the response to therapy of a given tumor. To date we are aware that the epigenetic and genomic landscapes of tumors which origin, histopathological diagnoses and clinical stages are almost identical can be highly heterogeneous. Initially, the Human Genome Project represented the reference map for the human genome and provided the ideal background for the development of technology and analytic tools to decipher and rationalize enormous quantities of genomic data [1]. Subsequently, the National Research Council reported on the requirement of a precise taxonomy of human disease based on the continuous flow of molecular data originating from the OMIC approaches. This led The Cancer Genome Atlas (TGCA) and the International Cancer Genome Consortium (ICGC) toward the molecular taxonomy of different human cancers. A large spectrum of gene mutations has been identified [1]. They can be categorized in: (a) ***passenger*** mutations that are the majority and may be biologically inactive and clinically irrelevant; (b) ***driver*** mutations whose activity is required for the aberrant growth, survival and chemoresistance of human cancers. Driver mutations have been the main molecular targets to be tackled with “smart” drugs, thus providing the rationale for precise medicine. Next Generation Sequence (NGS) technology has enabled to identify actionable targets such as EGFR in lung cancer and BRAF in melanoma [1,2]. Since these drugs benefit only those patients carrying specific driver mutations the identification of biomarkers that can predict treatment responses is vital for the success of the precise cancer therapy and for the development of anticancer drugs. EGFR mutations are considered biomarkers for selecting lung cancer patients for the treatment with EGFR inhibitors [3]. Gefinitib and erlotinib represent the first choice for the treatment of lung cancer patients carrying EGFR mutations and prolong significantly the progression-free survival of the selected patients. Despite it, both gefinitib and erlotinib cannot be used to treat all lung cancer patients harbouring EGFR mutations due to mutation site heterogeneity which negatively impacts on the affinity of EGFR inhibitors to the mutated EGFR and consequently of the efficacy of the treatment. Lung cancer patients develop resistance to EGFR inhibitors due mostly common (50% of EGFR mutated lung cancer patients) to additional EGFRT90M mutation [3]. Unlike EGFR, other ***driver*** mutations as those affecting the p53 gene, the most frequent target of genetic alterations in human cancers, have not yet led to the development of targeted drugs to be used in the treatment of human cancers carrying mutant p53 proteins [4]. This clearly says, that while thousands of cancer genome profiles have enormously improved the molecular taxonomy of human cancers, they have only paved a background for precise cancer therapy which urges to be continuously fed towards the identification of precise cancer biomarkers. The improvement of methodologies for the isolation of circulating tumoral DNA from patients enrolled in cancer genome-driven trials coupled with NGS might contribute to tailor more precisely cancer therapy [1]. At the same time, we have learned from the OMIC technologies that what so called non-coding portion of the human genome plays a fundamental role in regulating the expression and the activity of the genomic coding regions [5]. The last two decades have witnessed the identification of non-coding transcripts which accordingly to their respective lengths have been distinguished in long non-coding RNAs (lncRNAs), microRNAs, small interfering RNAs (siRNAs) and Piwi-interacting RNAs (piRNAs). MicroRNAs, which regulate gene expression at the posttranscriptional level either inhibiting translation or promoting degradation of target mRNAs, emerge to be powerful to distinguish tumor tissues from their matched surrounding non-tumoral samples, to classify tumor hystotypes, to predict tumor recurrence, to identify responders vs non-responders and to monitor response to cancer therapy [5,6,7]. MicroRNAs might represent early indicators of future breast cancer incidence. Previous evidence has shown that metabolic and environmental risk factors may alter the expression of microRNAs. MicroRNA profiling of the leucocytes of healthy pre-menopausal women recruited in the ORDET prospective cohort study over a follow-up period of 20 years revealed that microRNA downregulation represents a very early alteration in the development of breast cancer [8]. Selected microRNA alterations identified in ORDET were also found in different breast cancer databases, thus strengthening their value as early long-term predictors of breast cancer occurrence [8]. MicroRNAs can also be found in blood and other biological fluids as circulating factors lined into exosomial vesicles. Despite the molecular mechanisms underlying the production and the release from tumoral cells and the intrinsic processing occurring in the exosomes are yet underexplored their potential to unveil powerful and precise cancer biomarkers is certainly promising and might provide with an additional option to treat cancer successfully.
